# 2-(4-Phenyl-3*H*-1,5-benzodiazepin-2-yl)phenol

**DOI:** 10.1107/S1600536808034752

**Published:** 2008-11-08

**Authors:** Feng-Ke Yang, Wei Cheng, Yi-Ning Ding

**Affiliations:** a College of Chemical Engineering, Qingdao University of Science and Technology, Qingdao 266042, People’s Republic of China; b Key Laboratory of Advanced Materials, Qingdao University of Science and Technology, Qingdao 266042, People’s Republic of China

## Abstract

In the title compound, C_21_H_16_N_2_O, the dihedral angle between the pendant aromatic rings is 74.2–(1)°.. The conformation is stabilized by an intramolecular O—H⋯N hydrogen bond.

## Related literature

For the biological properties of Schiff bases, see: Abu-Hussen (2006 ▶); Mladenova *et al.* (2002 ▶); Singh *et al.* (2006 ▶). For the applications of nitro­gen heterocyclic compounds, see: Adsule *et al.* (2006 ▶). For bond-length data, see: Allen *et al.* (1987 ▶).
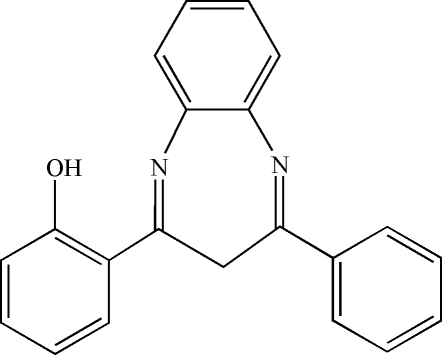


## Experimental

### 

#### Crystal data

C_21_H_16_N_2_O
*M* *_r_* = 312.36
Monoclinic, 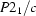

*a* = 6.3787 (13) Å
*b* = 16.695 (3) Å
*c* = 16.166 (4) Å
β = 110.72 (3)°
*V* = 1610.2 (7) Å^3^
*Z* = 4
Mo *K*α radiation
μ = 0.08 mm^−1^
*T* = 298 (2) K
0.20 × 0.20 × 0.10 mm

#### Data collection

Bruker SMART 1K CCD area-detector diffractometer
Absorption correction: none
6422 measured reflections
2806 independent reflections
1726 reflections with *I* > 2σ(*I*)
*R*_int_ = 0.061

#### Refinement

*R*[*F* ^2^ > 2σ(*F* ^2^)] = 0.099
*wR*(*F* ^2^) = 0.198
*S* = 1.18
2806 reflections
217 parameters
H-atom parameters constrained
Δρ_max_ = 0.22 e Å^−3^
Δρ_min_ = −0.17 e Å^−3^

### 

Data collection: *SMART* (Bruker, 2001 ▶); cell refinement: *SAINT* (Bruker, 2001 ▶); data reduction: *SAINT*; program(s) used to solve structure: *SHELXTL* (Sheldrick, 2008 ▶); program(s) used to refine structure: *SHELXTL*; molecular graphics: *SHELXTL*; software used to prepare material for publication: *SHELXTL* and local programs.

## Supplementary Material

Crystal structure: contains datablocks global, I. DOI: 10.1107/S1600536808034752/at2658sup1.cif

Structure factors: contains datablocks I. DOI: 10.1107/S1600536808034752/at2658Isup2.hkl

Additional supplementary materials:  crystallographic information; 3D view; checkCIF report

## Figures and Tables

**Table 1:** Hydrogen-bond geometry (Å, °)

*D*—H⋯*A*	*D*—H	H⋯*A*	*D*⋯*A*	*D*—H⋯*A*
O1—H1*A*⋯N2	0.83	1.82	2.563 (5)	147

Symmetry codes: (i) 
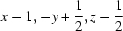
; (ii) 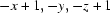; (iii) 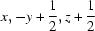.

**Fig. 1.**
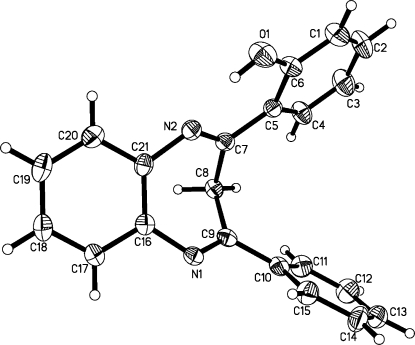
The structure of the title compound showing 30% probability displacement ellipsoids and the atom-numbering scheme.

## supplementary crystallographic information

### Comment

Monocondensed Schiff bases use as intermediates, in the synthesis of unsymmetrical multidentate Schiff base lingands. So they are attractive. Schiff bases often exhibit important biological activities such as antifungal (Singh *et al.*, 2006), antibacterial (Abu-Hussen *et al.*, 2006) and antitumor (Mladenova *et al.*, 2002). Nitrogen heterocyclic compounds have been used widely in the pharmaceutical industry, medicine and agriculture for their biological activity because of their antimicrobial, antipyretic, anti-inflammatory, and anticancer properties (Adsule *et al.*, 2006). In this paper, we have synthesized a new Schiff base compound by the condensation of 2-(4-phenyl-3*H*-benzo[*b*][1,4]diazepin-2-yl)-phenol with diaminobenzene and characterized it by X-ray crystallography.

All the bond lengths in the compound are within normal range (Allen *et al.*, 1987). The C9—N1 bond length is 1.289 (5) Å while C7—N2 bond length is 1.302 (5) Å that confirm they are both double bonds. Five atoms N2, C5, C7, C8 and C21 are in a plane(p1). Four atoms N1, C8, C9, C10 are in a plane(p2). The benzene ring C1—C6(p3), is approximately planar with its immediate substituent atoms C7 and O1 with a maximum deviation of 0.035 Å for O1.The benzene ring C10—C15(p4), is approximately planar with its immediate substituent atoms C9 with a maximum deviation of 0.038 Å for C9. The benzene ring C16—C21(p5). The dihedral angles formed by p1 with the p3, p4, p5 are 11.06, 80.49, 40.56°, respectively. The dihedral angles formed by p2 with the p3, p5 are 84.81, 42.56 °, respectively. The dihedral angles between p1 and p2 is 75.12 °. The molecular structure is stabilized by intramolecular O—H···N hydrogen-bonding interactions and the crystal structure is stabilized by C—H···π interactions {C13···*Cg*1 = 3.871, H13A···*Cg*1 = 3.161 Å, C13—H13A···*Cg*1 = 134.58° [Symmetry code: -1+x, 1/2-y, -1/2+z]; C18···*Cg*2 = 3.897, H18A···*Cg*2 = 3.126 Å, C18—H18A···*Cg*2 = 141.54° [Symmetry code: 1-x, -y, 1-z]; C19···*Cg*1 = 3.682, H19A···*Cg*1 = 3.169 Å, C19—H19A···*Cg*1 = 116.71° [Symmetry code: x, 1/2-y, 1/2+z]; C20···*Cg*2 = 3.685, H20A···*Cg*2 = 3.052 Å, C20—H20A···*Cg*2 = 126.85° [Symmetry code: x, 1/2-y, 1/2+z]. *Cg*1 and, *Cg*2 are the centroids of rings C1—C6, C10—C15, respectively}.

### Experimental

1-(2-Hydroxy-phenyl)-ethanone (13.6 g, 0.10 mol), chlorosyl-benzene (14.1 g, 0.10 mol), potassa (0.42 g) refluxed in absolute piperidine (15 ml) result in the yellow product of 1-(2-hydroxy-phenyl)-3-phenyl-propane-1,3-dione. The title compound was obtained by the reaction of 1-(2-hydroxy-phenyl)-3-phenyl-propane-1,3-dione (2.04 g, 0.01 mol) and benzene-1,2-diamine (1.08 g, 0.01 mol) without solvent. Single crystals suitable for X-ray measurements were obtained by slow evaporation of an absolute ethanol at room temperature.

### Refinement

H atoms were fixed geometrically to ride on their attached atoms, with C—H = 0.93–0.97 Å and O—H = 0.84 Å, and with *U*_iso_ =1.2*U*_eq_ (C) or 1.5 *U*_eq_ (O).

### Crystal data

**Table:**

C_21_H_16_N_2_O	*F*_000_ = 656
*M**_r_* = 312.36	*D*_x_ = 1.288 Mg m^−^^3^
Monoclinic, *P*2_1_/*c*	Mo *K*α radiation λ = 0.71073 Å
Hall symbol: -P 2ybc	Cell parameters from 2787 reflections
*a* = 6.3787 (13) Å	θ = 2.5–26.0º
*b* = 16.695 (3) Å	µ = 0.08 mm^−^^1^
*c* = 16.166 (4) Å	*T* = 298 (2) K
β = 110.72 (3)º	Block, yellow
*V* = 1610.2 (7) Å^3^	0.20 × 0.20 × 0.10 mm
*Z* = 4	

### Data collection

**Table:**

Bruker SMART 1K CCD area-detector diffractometer	1726 reflections with *I* > 2σ(*I*)
Radiation source: fine-focus sealed tube	*R*_int_ = 0.061
Monochromator: graphite	θ_max_ = 25.0º
*T* = 298(2) K	θ_min_ = 1.8º
Thin–slice ω scans	*h* = −7→7
Absorption correction: none	*k* = −9→19
6422 measured reflections	*l* = −19→19
2806 independent reflections	

### Refinement

**Table:**

Refinement on *F*^2^	Secondary atom site location: difference Fourier map
Least-squares matrix: full	Hydrogen site location: inferred from neighbouring sites
*R*[*F*^2^ > 2σ(*F*^2^)] = 0.099	H-atom parameters constrained
*wR*(*F*^2^) = 0.198	*w* = 1/[σ^2^(*F*_o_^2^) + (0.0623*P*)^2^ + 0.3681*P*] where *P* = (*F*_o_^2^ + 2*F*_c_^2^)/3
*S* = 1.18	(Δ/σ)_max_ < 0.001
2806 reflections	Δρ_max_ = 0.22 e Å^−^^3^
217 parameters	Δρ_min_ = −0.17 e Å^−^^3^
Primary atom site location: structure-invariant direct methods	Extinction correction: none

### Special details

**Table:**

Geometry. All e.s.d.'s (except the e.s.d. in the dihedral angle between two l.s. planes) are estimated using the full covariance matrix. The cell e.s.d.'s are taken into account individually in the estimation of e.s.d.'s in distances, angles and torsion angles; correlations between e.s.d.'s in cell parameters are only used when they are defined by crystal symmetry. An approximate (isotropic) treatment of cell e.s.d.'s is used for estimating e.s.d.'s involving l.s. planes.
Refinement. Refinement of *F*^2^ against ALL reflections. The weighted *R*-factor *wR* and goodness of fit *S* are based on *F*^2^, conventional *R*-factors *R* are based on *F*, with *F* set to zero for negative *F*^2^. The threshold expression of *F*^2^ > σ(*F*^2^) is used only for calculating *R*-factors(gt) *etc*. and is not relevant to the choice of reflections for refinement. *R*-factors based on *F*^2^ are statistically about twice as large as those based on *F*, and *R*- factors based on ALL data will be even larger.

### Fractional atomic coordinates and isotropic or equivalent isotropic displacement parameters (Å^2^)

**Table:**

	*x*	*y*	*z*	*U*_iso_*/*U*_eq_	
O1	0.0166 (5)	−0.38550 (18)	−0.5523 (2)	0.0622 (10)	
H1A	0.0571	−0.3466	−0.5754	0.075*	
N1	0.3086 (5)	−0.10096 (19)	−0.5027 (2)	0.0366 (9)	
N2	0.0328 (6)	−0.2397 (2)	−0.5993 (2)	0.0407 (9)	
C1	−0.2099 (8)	−0.4154 (3)	−0.4699 (3)	0.0593 (14)	
H1C	−0.1742	−0.4692	−0.4718	0.071*	
C2	−0.3515 (9)	−0.3930 (3)	−0.4277 (3)	0.0676 (16)	
H2B	−0.4122	−0.4317	−0.4013	0.081*	
C3	−0.4051 (9)	−0.3140 (4)	−0.4237 (3)	0.0678 (16)	
H3A	−0.5027	−0.2988	−0.3953	0.081*	
C4	−0.3130 (7)	−0.2574 (3)	−0.4623 (3)	0.0511 (13)	
H4A	−0.3491	−0.2039	−0.4588	0.061*	
C5	−0.1671 (7)	−0.2770 (3)	−0.5064 (3)	0.0373 (11)	
C6	−0.1186 (7)	−0.3587 (3)	−0.5099 (3)	0.0456 (12)	
C7	−0.0739 (7)	−0.2161 (3)	−0.5483 (3)	0.0367 (11)	
C8	−0.0850 (7)	−0.1287 (2)	−0.5304 (3)	0.0377 (11)	
H8A	−0.1908	−0.1183	−0.5008	0.045*	
H8B	−0.1287	−0.0980	−0.5848	0.045*	
C9	0.1498 (7)	−0.1083 (2)	−0.4713 (3)	0.0351 (10)	
C10	0.2076 (7)	−0.1005 (2)	−0.3739 (3)	0.0390 (11)	
C11	0.0546 (8)	−0.0730 (3)	−0.3376 (3)	0.0517 (13)	
H11A	−0.0883	−0.0578	−0.3744	0.062*	
C12	0.1128 (10)	−0.0680 (3)	−0.2474 (4)	0.0631 (15)	
H12A	0.0095	−0.0481	−0.2240	0.076*	
C13	0.3185 (10)	−0.0916 (3)	−0.1916 (3)	0.0651 (15)	
H13A	0.3551	−0.0891	−0.1307	0.078*	
C14	0.4708 (8)	−0.1193 (3)	−0.2274 (3)	0.0614 (15)	
H14A	0.6126	−0.1350	−0.1901	0.074*	
C15	0.4170 (8)	−0.1242 (3)	−0.3174 (3)	0.0515 (13)	
H15A	0.5218	−0.1435	−0.3404	0.062*	
C16	0.2675 (6)	−0.1189 (3)	−0.5918 (3)	0.0350 (10)	
C17	0.3790 (7)	−0.0723 (3)	−0.6349 (3)	0.0421 (11)	
H17A	0.4668	−0.0294	−0.6054	0.050*	
C18	0.3612 (8)	−0.0887 (3)	−0.7201 (3)	0.0550 (13)	
H18A	0.4321	−0.0558	−0.7486	0.066*	
C19	0.2394 (8)	−0.1533 (3)	−0.7638 (3)	0.0573 (14)	
H19A	0.2287	−0.1643	−0.8215	0.069*	
C20	0.1333 (8)	−0.2020 (3)	−0.7224 (3)	0.0543 (13)	
H20A	0.0558	−0.2469	−0.7515	0.065*	
C21	0.1407 (7)	−0.1845 (3)	−0.6372 (3)	0.0391 (11)	

### Atomic displacement parameters (Å^2^)

**Table:**

	*U*^11^	*U*^22^	*U*^33^	*U*^12^	*U*^13^	*U*^23^
O1	0.077 (2)	0.041 (2)	0.084 (3)	0.0033 (18)	0.047 (2)	−0.0025 (18)
N1	0.037 (2)	0.030 (2)	0.042 (2)	0.0003 (16)	0.0141 (17)	0.0018 (17)
N2	0.047 (2)	0.039 (2)	0.040 (2)	−0.0006 (18)	0.0201 (18)	−0.0035 (17)
C1	0.066 (3)	0.042 (3)	0.072 (4)	0.000 (3)	0.028 (3)	0.003 (3)
C2	0.077 (4)	0.060 (4)	0.078 (4)	0.001 (3)	0.043 (3)	0.021 (3)
C3	0.071 (4)	0.069 (4)	0.081 (4)	0.003 (3)	0.049 (3)	0.011 (3)
C4	0.055 (3)	0.046 (3)	0.060 (3)	0.007 (2)	0.030 (3)	0.010 (2)
C5	0.037 (3)	0.039 (3)	0.037 (3)	−0.005 (2)	0.014 (2)	−0.002 (2)
C6	0.044 (3)	0.040 (3)	0.055 (3)	−0.005 (2)	0.021 (2)	0.001 (2)
C7	0.030 (2)	0.038 (3)	0.037 (3)	−0.001 (2)	0.007 (2)	0.001 (2)
C8	0.038 (3)	0.036 (3)	0.043 (3)	0.003 (2)	0.020 (2)	0.002 (2)
C9	0.038 (2)	0.019 (2)	0.048 (3)	0.0065 (19)	0.015 (2)	0.002 (2)
C10	0.055 (3)	0.022 (2)	0.047 (3)	0.002 (2)	0.026 (2)	−0.004 (2)
C11	0.060 (3)	0.045 (3)	0.053 (3)	0.007 (2)	0.025 (3)	−0.005 (2)
C12	0.084 (4)	0.061 (4)	0.058 (4)	0.000 (3)	0.041 (3)	−0.011 (3)
C13	0.094 (4)	0.066 (4)	0.041 (3)	−0.020 (3)	0.030 (3)	−0.010 (3)
C14	0.062 (3)	0.087 (4)	0.033 (3)	−0.006 (3)	0.014 (3)	0.003 (3)
C15	0.050 (3)	0.057 (3)	0.050 (3)	0.002 (3)	0.020 (2)	0.001 (2)
C16	0.030 (2)	0.037 (3)	0.037 (3)	0.009 (2)	0.010 (2)	0.005 (2)
C17	0.042 (3)	0.043 (3)	0.044 (3)	0.003 (2)	0.019 (2)	0.008 (2)
C18	0.065 (3)	0.060 (4)	0.044 (3)	0.002 (3)	0.024 (3)	0.012 (3)
C19	0.064 (3)	0.074 (4)	0.041 (3)	0.009 (3)	0.027 (3)	0.000 (3)
C20	0.061 (3)	0.058 (4)	0.047 (3)	−0.007 (3)	0.023 (2)	−0.010 (3)
C21	0.039 (3)	0.045 (3)	0.035 (3)	0.006 (2)	0.015 (2)	0.002 (2)

### Geometric parameters (Å, °)

**Table:**

O1—C6	1.353 (5)	C10—C11	1.382 (6)
O1—H1A	0.8347	C10—C15	1.383 (6)
N1—C9	1.289 (5)	C11—C12	1.375 (6)
N1—C16	1.402 (5)	C11—H11A	0.9300
N2—C7	1.302 (5)	C12—C13	1.361 (7)
N2—C21	1.414 (5)	C12—H12A	0.9300
C1—C2	1.363 (6)	C13—C14	1.375 (6)
C1—C6	1.385 (6)	C13—H13A	0.9300
C1—H1C	0.9300	C14—C15	1.374 (6)
C2—C3	1.371 (6)	C14—H14A	0.9300
C2—H2B	0.9300	C15—H15A	0.9300
C3—C4	1.372 (6)	C16—C17	1.396 (5)
C3—H3A	0.9300	C16—C21	1.404 (6)
C4—C5	1.397 (6)	C17—C18	1.369 (6)
C4—H4A	0.9300	C17—H17A	0.9300
C5—C6	1.405 (6)	C18—C19	1.370 (6)
C5—C7	1.459 (6)	C18—H18A	0.9300
C7—C8	1.495 (5)	C19—C20	1.373 (6)
C8—C9	1.503 (5)	C19—H19A	0.9300
C8—H8A	0.9700	C20—C21	1.392 (5)
C8—H8B	0.9700	C20—H20A	0.9300
C9—C10	1.491 (5)		
			
C6—O1—H1A	108.9	C15—C10—C9	119.5 (4)
C9—N1—C16	119.8 (4)	C12—C11—C10	120.3 (5)
C7—N2—C21	121.4 (4)	C12—C11—H11A	119.9
C2—C1—C6	120.6 (5)	C10—C11—H11A	119.9
C2—C1—H1C	119.7	C13—C12—C11	121.4 (5)
C6—C1—H1C	119.7	C13—C12—H12A	119.3
C1—C2—C3	120.5 (5)	C11—C12—H12A	119.3
C1—C2—H2B	119.7	C12—C13—C14	118.5 (5)
C3—C2—H2B	119.7	C12—C13—H13A	120.7
C2—C3—C4	119.1 (5)	C14—C13—H13A	120.7
C2—C3—H3A	120.4	C15—C14—C13	121.1 (5)
C4—C3—H3A	120.4	C15—C14—H14A	119.5
C3—C4—C5	122.8 (5)	C13—C14—H14A	119.5
C3—C4—H4A	118.6	C14—C15—C10	120.2 (4)
C5—C4—H4A	118.6	C14—C15—H15A	119.9
C4—C5—C6	116.3 (4)	C10—C15—H15A	119.9
C4—C5—C7	121.9 (4)	C17—C16—N1	116.8 (4)
C6—C5—C7	121.7 (4)	C17—C16—C21	118.3 (4)
O1—C6—C1	117.4 (4)	N1—C16—C21	124.6 (4)
O1—C6—C5	122.0 (4)	C18—C17—C16	121.0 (4)
C1—C6—C5	120.6 (4)	C18—C17—H17A	119.5
N2—C7—C5	118.3 (4)	C16—C17—H17A	119.5
N2—C7—C8	119.3 (4)	C17—C18—C19	120.4 (5)
C5—C7—C8	122.3 (4)	C17—C18—H18A	119.8
C7—C8—C9	103.8 (3)	C19—C18—H18A	119.8
C7—C8—H8A	111.0	C18—C19—C20	120.2 (5)
C9—C8—H8A	111.0	C18—C19—H19A	119.9
C7—C8—H8B	111.0	C20—C19—H19A	119.9
C9—C8—H8B	111.0	C19—C20—C21	120.5 (5)
H8A—C8—H8B	109.0	C19—C20—H20A	119.8
N1—C9—C10	118.2 (4)	C21—C20—H20A	119.8
N1—C9—C8	121.2 (4)	C20—C21—C16	119.5 (4)
C10—C9—C8	120.6 (4)	C20—C21—N2	116.2 (4)
C11—C10—C15	118.5 (4)	C16—C21—N2	124.1 (4)
C11—C10—C9	122.0 (4)		
			
C6—C1—C2—C3	−0.3 (8)	N1—C9—C10—C15	−31.4 (6)
C1—C2—C3—C4	−0.5 (8)	C8—C9—C10—C15	145.4 (4)
C2—C3—C4—C5	0.6 (8)	C15—C10—C11—C12	1.4 (7)
C3—C4—C5—C6	0.2 (7)	C9—C10—C11—C12	178.7 (4)
C3—C4—C5—C7	178.9 (4)	C10—C11—C12—C13	−1.7 (8)
C2—C1—C6—O1	−178.5 (5)	C11—C12—C13—C14	1.3 (8)
C2—C1—C6—C5	1.1 (7)	C12—C13—C14—C15	−0.8 (8)
C4—C5—C6—O1	178.6 (4)	C13—C14—C15—C10	0.6 (7)
C7—C5—C6—O1	−0.1 (6)	C11—C10—C15—C14	−0.8 (7)
C4—C5—C6—C1	−1.0 (6)	C9—C10—C15—C14	−178.3 (4)
C7—C5—C6—C1	−179.7 (4)	C9—N1—C16—C17	145.2 (4)
C21—N2—C7—C5	−175.6 (3)	C9—N1—C16—C21	−41.1 (6)
C21—N2—C7—C8	0.7 (6)	N1—C16—C17—C18	175.7 (4)
C4—C5—C7—N2	−169.8 (4)	C21—C16—C17—C18	1.6 (6)
C6—C5—C7—N2	8.8 (6)	C16—C17—C18—C19	−2.4 (7)
C4—C5—C7—C8	14.0 (6)	C17—C18—C19—C20	0.3 (7)
C6—C5—C7—C8	−167.4 (4)	C18—C19—C20—C21	2.5 (7)
N2—C7—C8—C9	−71.1 (5)	C19—C20—C21—C16	−3.3 (7)
C5—C7—C8—C9	105.0 (4)	C19—C20—C21—N2	−177.9 (4)
C16—N1—C9—C10	171.1 (4)	C17—C16—C21—C20	1.3 (6)
C16—N1—C9—C8	−5.7 (6)	N1—C16—C21—C20	−172.3 (4)
C7—C8—C9—N1	75.8 (4)	C17—C16—C21—N2	175.4 (4)
C7—C8—C9—C10	−100.9 (4)	N1—C16—C21—N2	1.8 (6)
N1—C9—C10—C11	151.3 (4)	C7—N2—C21—C20	−143.7 (4)
C8—C9—C10—C11	−31.9 (6)	C7—N2—C21—C16	42.0 (6)

### Hydrogen-bond geometry (Å, °)

**Table:**

*D*—H···*A*	*D*—H	H···*A*	*D*···*A*	*D*—H···*A*
O1—H1A···N2	0.83	1.82	2.563 (5)	147
C13—H13A···Cg1^i^	0.93	3.16	3.871	135
C18—H18A···Cg2^ii^	0.93	3.13	3.897	142
C19—H19A···Cg1^iii^	0.93	3.17	3.682	117
C20—H20A···Cg2^iii^	0.93	3.05	3.685	127

Symmetry codes: (i) *x*−1, −*y*+1/2, *z*−1/2; (ii) −*x*+1, −*y*, −*z*+1; (iii) *x*, −*y*+1/2, *z*+1/2.
